# Hypoxic responsiveness and gut fermentation capacity in heart failure patients: preliminary results

**DOI:** 10.3389/fphys.2025.1728753

**Published:** 2026-01-08

**Authors:** Aleksandra Mikołajczak, Rafał Seredyński, Marzena Gonerska, Mateusz Sokolski, Bartłomiej Paleczny, Beata Ponikowska

**Affiliations:** 1 Department of Physiology and Pathophysiology, Wroclaw Medical University, Wroclaw, Poland; 2 Institute of Heart Diseases, Wroclaw Medical University, Wroclaw, Poland

**Keywords:** gut microbiota, heart failure, hydrogen breath test, peripheral chemoreceptors, transient hypoxia test

## Abstract

**Background:**

The gut microbiota has emerged as a key contributor to cardiovascular regulation. Acute stimulation of microbial fermentation with lactulose enhances hypoxic ventilatory response (HVR) in healthy subjects, indicating increased peripheral chemoreceptor (PCh) responsiveness. Given that heart failure (HF) is characterized by PCh hyperactivity, this study investigated whether enhancing intestinal fermentation could acutely modify chemoreceptor-driven responses in HF patients.

**Methods:**

HF patients (n = 12; all males; age: 59.2[15.8]y; 67% in NYHA III) underwent transient hypoxia test twice: before and ∼120 min after ingesting a gut-fermentation-stimulating meal. Hydrogen in expired air was measured repeatedly and used to stratify the patients into high early fermentation (HEF) and low early fermentation (LEF) groups. Ventilatory (HVR) and cardiovascular (heart rate, blood pressure, systemic vascular resistance) responses to hypoxia were measured.

**Results:**

HEF patients, as compared with the LEF group, displayed: (1) higher pre-lactulose HVR (mean ± SD, L/min/SpO_2_: 0.680 ± 0.284 vs. 0.343 ± 0.122; p = 0.024), (2) pre- and post-lactulose SVR response (mean ± SD, dyn s/cm^5^/SpO_2_: for pre-lactulose comparison, 35.40 ± 24.41 vs. 9.96 ± 1.80, p = 0.039; for post-lactulose comparison, 37.19 ± 25.75 vs. 9.22 ± 4.33, p = 0.026). HVR in the HEF group correlated with the net hydrogen excretion during the lactulose test (r = 0.85, p = 0.033).

**Conclusion:**

Our preliminary results, derived from a small, uncontrolled physiological experiment conducted in 12 H F patients, imply a link between the upper gut microbial fermentation capacity and the baseline peripheral chemoreflex sensitivity in this population. Given the exploratory and non-randomized design, these findings should be interpreted with caution, and larger controlled studies are needed to confirm the nature and clinical relevance of this association.

## Introduction

The gastrointestinal microbiota has emerged as a key contributor to numerous physiological processes and displays remarkable adaptability to environmental and metabolic challenges. It can be regarded as a discrete organ of substantial plasticity, capable of influencing distant organs through the release of bioactive metabolites (Marques et al., 2018; Fujisaka et al., 2023). Among its systemic effects, the gut microbiota plays an important role in cardiovascular regulation (Tang et al., 2017; Marques et al., 2018). Fermentation-derived short-chain fatty acids, such as acetate and propionate, can modulate vascular tone via G-protein-coupled receptors (e.g., GPR41, GPR43, Olfr78), thereby affecting blood pressure and peripheral resistance. These same metabolites and other microbially derived compounds have also been suggested to influence cardiovascular control indirectly by altering the sympathovagal balance and autonomic reflexes (Toral et al., 2019; Muller et al., 2020).

In line with this concept, we have recently demonstrated in healthy subjects that acute stimulation of gut microbial fermentation with lactulose enhances the ventilatory response to transient hypoxia, reflecting a transient increase in peripheral chemoreflex sensitivity (Seredyński et al., 2021). This finding suggests that fermentation-related signalling can presumably modulate carotid body function and, ultimately, the autonomic cardiovascular control.

Sympathetic overactivation and the altered cardiopulmonary reflexes (hyperactive peripheral chemoreceptors [PCh] in particular) are hallmarks of the chronic heart failure (HF) syndrome and are directly linked to adverse outcomes (Ponikowski et al., 2001; Giannoni et al., 2023). Given the postulated interaction between gut microbial activity and chemoreflex function in healthy humans, together with well-documented alterations in gut microbiota composition and barrier integrity in HF (Tang et al., 2017), it appears reasonable to explore whether enhancing intestinal fermentation can acutely modify chemoreceptor-driven ventilatory responses in this patient population.

In this paper, we attempted to translate our findings from healthy subjects to HF patients. In brief, chronic HF patients underwent the assessment of PCh function with the transient hypoxia test twice, before and ∼120 min after the ingestion of a gut-fermentation-stimulating meal. The content of hydrogen in the expired air was assessed repeatedly during the experiment and taken as a measure of fermentation intensity.

## Materials and methods

### Study population and ethical approval

Patients with symptomatic, stable HF in NYHA class II-III, aged ≥18 years, hospitalized at the Institute of Heart Diseases, University Hospital in Wroclaw (Poland) were invited to participate in the study. Exclusion criteria were as follows: (i) inability to undergo PCh assessment with the transient hypoxia test due to dyspnea, (ii) antibiotic therapy within 1 month prior to enrollment, (iii) inflammatory bowel disease, (iv) respiratory tract infection within 1 month prior to enrollment, (v) use of laxatives or medications affecting intestinal motility within 1 week prior to enrollment.

The protocol of the study was approved by the Bioethics Committee of Wroclaw Medical University (approval no. KB-827/2021). All patients provided written informed consent. The study adhered to the principles of the Declaration of Helsinki, with the exception of database registration.

### Study protocol

All assessments were conducted at the Institute of Heart Diseases, in a dedicated physiological testing room, maintained at a stable temperature of 22 °C–24 °C, with dim lighting and minimal noise. The protocol previously used in healthy subjects (Seredyński et al., 2021) was adopted for the present study. Patients were tested following overnight fasting. They underwent the PCh function assessment with the transient hypoxia test twice, before and >120 min after ingestion of a meal containing 10 g of lactulose (15 mL of Duphalac Lactulosum, Abbott Ltd., Montreal, Canada) dissolved in 200 mL of water. The first hypoxia test has started after 15 min of resting. The 10-minute-long segment of the resting recording was used to estimate the resting values of the ventilatory and hemodynamic parameters.

Hydrogen breath test (HBT) was performed before the lactulose meal (the basal breath) and at 15, 30, 45, 60 and 120 min thereafter. The content of hydrogen in the exhaled air (H_2_, ppm) was measured with a handheld device (Gastrolyser Gastro+, Bedfont Scientific, Ltd., Maidstone, United Kingdom). Prior to the basal breath, patients rinsed their mouth with a mouthwash containing chlorhexidine (Eludril, Pierre Fabre Oral Care, Paris, France). Subsequently, the patient was asked to perform a prolonged exhalation through the analyser.

Blood glucose concentration (mg/mL) was measured before the test meal (alongside the basal breath) and right after the second transient hypoxia test. The Accu-Check Active glucometer and dedicated test strips (Roche Diabetes Care, Inc., Vienna, Austria) were used to measure blood glucose level in the fingertip blood.

#### Safety precautions

Since its introduction in 1973 (Edelman et al., 1973), the transient hypoxia test has been extensively validated in chronic HF populations (Ponikowski et al., 2001; Niewinski et al., 2013; Kulej-Lyko et al., 2022), with previous studies consistently demonstrating that the method is safe, well-tolerated, and associated with no major adverse events. In the present study, all assessments were performed in the physiological testing room of the Institute of Heart Diseases, Wroclaw Medical University, located within the clinical area of the Department of Cardiology and in direct proximity to the Emergency Department. A cardiologist was always present throughout each examination. Patient safety was ensured by continuous real-time monitoring of ECG, blood oxygen saturation, non-invasive finger arterial blood pressure, respiratory airflow, and end-tidal CO_2_. Participants were instructed to signal any alarming symptoms (e.g., intense or prolonged breathlessness, sudden weakness, palpitations, unusual discomfort) by raising a hand, prompting immediate termination of the test. The operator carefully observed the patient during the entire protocol, with particular attention during and immediately after each nitrogen administration. Predefined stopping rules included: (i) patient request; (ii) loss of mask tolerance; (iii) arrhythmia or concerning ECG changes; (iv) excessive or poorly tolerated desaturation; or (v) any clinical concern raised by the supervising cardiologist.

### The study equipment

The experimental setup for the transient hypoxia test used in our laboratory has been described in detail elsewhere (Niewinski et al., 2013; Niewinski et al., 2017). In brief, the breathing circuit consists of a facial mask (7450 V2 mask, Hans Rudolph, Inc., Shawnee, KS, United States) connected to a two-way non-rebreathing valve (Hans Rudolph, Inc.), and two breathing tubes: an inspiratory arm for nitrogen administration and an expiratory arm connected to a 1000 L/min flowhead of a differential pressure transducer (FE141 Spirometer, ADInstruments, Dunedin, New Zealand). Expiratory airflow was measured continuously, and instantaneous minute ventilation (VI, L/min) was calculated based on tidal volume (VT, L/min) and breathing frequency (breaths/min). Blood oxygen saturation (SpO_2_, %) and the end-tidal CO_2_ (etCO_2_, mmHg) were measured with a pulse oximeter (Radical-7, Masimo Corporation, Irvine, CA, United States; ear probe), and a capnograph (Capstar 100, CWE, Ardmore, PL, United States), respectively.

Finger blood pressure was recorded continuously with photoplethysmography (BMEYE, Amsterdam, Netherlands), and used to calculate systolic blood pressure (SBP, mmHg), diastolic blood pressure (DBP), mean arterial pressure (MAP, mmHg), and systemic vascular resistance (SVR, dyn s/cm^5^). Heart rate (bpm) was derived from the ECG signal (BioAmp, ADInstruments).

All physiological data were recorded continuously, digitized at 1 kHz (PowerLab 16/30, ADInstruments) and saved on a laptop.

### The transient hypoxia test

Standard transient hypoxia test was used to assess the function of PCh (Edelman et al., 1973; Niewinski et al., 2013; Seredyński et al., 2021). Contrary to the other hypoxia-based protocols, i.e., steady-state test, the transient hypoxia has been suggested to isolate the pure PCh response rather than a broader systemic reaction to sustained hypoxia (Pfoh et al., 2016). The test is performed with the patient lying supine and involves repeated episodes of brief hypoxia produced by filling the breathing circuit with pure nitrogen. The entire test includes 4–12 episodes of hypoxia, each lasting up to ∼40 s, interspersed with ≥3 min periods of room-air breathing. The first exposure lasted 5 s, and the duration of subsequent ones was gradually prolonged until either a nadir SpO_2_ of approximately 70% was reached or a distinct and reproducible rise in V was observed. Thereafter, the duration of nitrogen administrations was randomly varied within the previously determined range to produce a broad spectrum of SpO_2_ nadirs, while avoiding excessive or poorly tolerated discomfort.

### Data processing and statistical analysis

The Kolmogorov-Smirnov was used to verify the normality of data distribution. Continuous variables were presented as mean with standard deviation (SD) or median with lower and upper quartile, for data with normal- and non-normal distribution, respectively. Qualitative data were shown as numbers and percentages. Within-subject and between-subject comparisons were conducted using parametric tests (paired and unpaired Student’s t-test, respectively) or nonparametric alternatives (the Wilcoxon test and the U Mann-Whitney test, respectively). Welch’s t-test was applied for the between-group comparisons with unequal variances, and Pearson’s linear correlation coefficient was used to investigate associations between variables. Effect sizes for variables demonstrating statistically significant between-group differences were quantified using Cohen’s *d* and 95% confidence intervals (95%CI) around the mean were additionally reported. LabChart (ADInstruments), Statistica v.13.3 (StatSoft, Tulsa, OK, United States) and MATLAB R2021b (Mathworks, Natick, MA, United States) were used for data processing and statistical analysis. A p-value <0.05 was considered significant.

The results of the transient hypoxia test and the hydrogen breath test were processed and analyzed independently by two researchers (B. Paleczny and R. Seredyński, respectively), who did not communicate with each other regarding these analyses.

#### The transient hypoxia test–hypoxic ventilatory response

The computational procedure for assessing the hypoxic ventilatory response (HVR) has been described previously (Niewinski et al., 2013; Paleczny et al., 2019; Seredyński et al., 2021). Briefly, HVR (L/min/SpO_2_) was defined as the slope of the regression line relating ventilation (V) to arterial oxygen saturation (SpO_2_) during transient hypoxia. For each nitrogen-induced hypoxic episode, two data points were plotted: (i) a pre-hypoxic point, representing the mean SpO_2_ and corresponding mean V over 60 s before hypoxia, and (ii) a post-hypoxic point, defined by the SpO_2_ nadir (within 1 min after N_2_ administration) and the mean of the three highest consecutive V values obtained within 5 beats before to 20 beats after the nadir.

If the quality of the respiratory tracing (VI) substantially deteriorated within the analysis window, the corresponding hypoxic episode was excluded from further analysis of HVR and cardiovascular responses (see below).

#### The transient hypoxia test–cardiovascular responses

HR, SBP, MAP, and SVR data were converted to beat-to-beat format and analyzed analogously to HVR. For each hypoxic episode, two points were plotted using the corresponding pre- and post-hypoxic SpO_2_ values. Post-hypoxic values were defined as follows: the highest HR, SBP, and MAP, and the lowest SVR observed within the same analysis window (5 beats before to 20 beats after the SpO_2_ nadir). Slopes of the resulting regression lines quantified the magnitude of the cardiovascular responses to transient hypoxia: Hyp-HR (bpm/SpO_2_), Hyp-SBP slope (mmHg/SpO_2_), Hyp-MAP slope (mmHg/SpO_2_), and Hyp-SVR slope (dyn s/cm^5^/SpO_2_).

#### The hydrogen breath test

Patients with an exhaled H_2_ rise >20 ppm (above baseline value) within 60 min after 10 g lactulose ingestion were classified to the High Early microbial Fermentation (HEF) group (in contrast to the Low Early microbial Fermentation, LEF).

HEF classification criteria were based on the previously proposed protocols for a small intestinal bacterial overgrowth (SIBO) diagnosis, designed to reduce confounding from colonic fermentation and oro-cecal transit variability and to minimize the risk of false-positive results (Sunny et al., 2016; Hammer et al., 2022; Dahlgren et al., 2025).

Net hydrogen excretion (net H_2_) was calculated as the difference between the hydrogen excretion level at each time point (between 15 and 120 min) and the hydrogen excretion level in the basal breath. Cumulative net H_2_ measurements over time (15–120 min) were integrated into a single measurement by the area under the concentration receiver operating characteristic curve (net H_2_ AUC) with MATLAB R2021b software (Mathworks, Natick, MA, United States) using the trapezoid method.

## Results

Sixteen HF patients were recruited to the study. Four participants were excluded from further analyses due to the incomplete data (2 participants; measurements were terminated due to the reported discomfort, e.g., related to the pressure of the oronasal mask) or low quality recordings (2 participants; data could not be reliably computed due to the periodic breathing and fluctuations in the blood oxygen saturation). Baseline characteristics of 12 patients (all males) included are summarized in Table 1.

**TABLE 1:** Basic characteristics, resting physiological variables and hypoxic responses of the study participants (n = 12).

Basic characteristics [mean (SD) or median [Q1 – Q3] or n (%)]	
Age [years]	59.2 (15.8)
Male, [n (%)]	12 (100%)
NYHA class III [n (%)]	8 (67)
LVEF [%]	23.5 (7.5)
Hemoglobin [g/dL]	15.1 (2.1)
Creatinine [mg/dL]	1.27 [0.96–1.36]
Sodium [mmol/L]	137 [136–139]
Potassium [mmol/L]	4.4 [4.0–4.9]
Hemoglobin A1C [%]	6.2 (0.5)
NT-proBNP [pg/mL]	2419.1 [1068.1–6267.4]
Beta-blocker [n (%)]	12 (100)
ACE-I/ARB/ARNI [n (%)]	11 (92)
MRA [n (%)]	12 (100)
SGLT2i [n (%)]	11 (92)
Loop diuretics [n (%)]	11 (92)
Thiazides [n (%)]	0 (0)
CCB [n (%)]	0 (0)
Statin [n (%)]	5 (58)

Resting parameters	Pre-lactulose [mean (SD)] or median [Q1 – Q3]	Post-lactulose [mean (SD)]
VI [L/min]	12.8 (3.09)	14.6 (4.88)
VT [L]	0.81 (0.22)	0.91 (0.28)
HR [beats/min]	69.1 (8.51)	68.4 (7.76)
SBP [mmHg]	104.0 (17.82)	103.4 (14.91)
DBP [mmHg]	63.5 [57.17–67.85]	62.3 (6.18)
MAP [mmHg]	77.0 (11.41)	75.8 (8.39)
SVR [dyn s/cm^5^]	1449.9 (495.94)	1328.1 (503.29)
Blood glucose [mg/dL]	96.7 (10.12)	92.5 (8.78)

Hypoxic responses	Pre-lactulose		Post-lactulose	
	Mean (SD)	r (SD)	Mean (SD)	r (SD)
HVR [L/min/SpO_2_]	−0.51 (0.37)	0.78 (0.11)	−0.71 (0.44)	0.80 (0.14)
Hyp-HR [beats/min/SpO_2_]	−0.88 (0.53)	0.79 (0.12)	−0.92 (0.67)	0.77 (0.12)
Hyp-SBP [mmHg/SpO_2_]	−0.92 (0.54)	0.80 (0.10)	−0.96 (0.59)	0.78 (0.10)
Hyp-MAP [mmHg/SpO_2_]	−0.59 (0.26)	0.79 (0.11)	0.63 (0.29)	0.76 (0.10)
Hyp-SVR [dyn s/cm^5^/SpO_2_]	22.68 (19.18)	0.75 (0.13)	23.21 (18.10)	0.78 (0.11)

Resting variables and hypoxic responses were evaluated twice during each test (before and ∼2 h after the test meal–pre- and post-lactulose, respectively). Hypoxic responses are represented by slopes of the linear relationship between SpO_2_ and the given ventilatory or hemodynamic parameter. SD, standard deviation; NYHA, class, New York Heart Association functional class; LVEF, left ventricular ejection fraction; NT-proBNP, N-terminal pro-B-type natriuretic peptide; ACE-I, angiotensin-converting enzyme inhibitor; ARB, angiotensin receptor blocker; ARNI, angiotensin receptor/nephrilysin inhibitor; MRA, mineralocorticoid receptor antagonist; SGLT2i, sodium glucose co-transporter type 2 inhibitor; CCB, calcium channel blocker; DBP, diastolic blood pressure; HR, heart rate; HVR, hypoxic ventilatory response; MAP, mean arterial pressure; SBP, systolic blood pressure; SVR, systemic vascular resistance; VI, minute ventilation; VT, tidal volume.

### Hydrogen excretion

**FIGURE 1:**
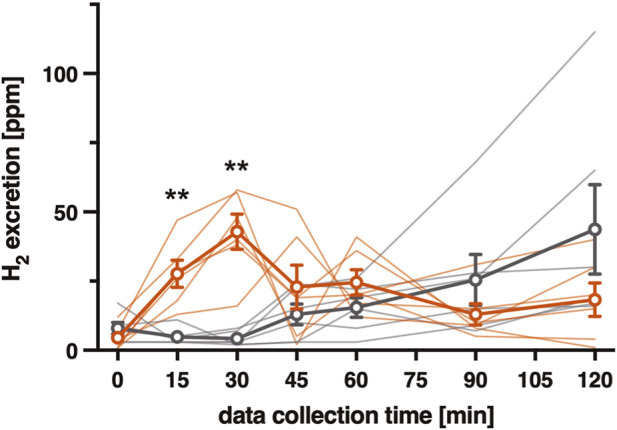
Hydrogen content in the exhaled air (parts per million, ppm), recorded before (time 0) and after (time 15–120) the lactulose ingestion. Circles and thick lines represent mean values for HEF (red) and LEF (grey) groups; error bars represent ±SEM. Thin lines represent individual data of 12 study participants. (**) HEF vs. LEF mean values comparison, p < 0.01 according to the Welch’s t-test. HEF, high early (microbial) fermentation; LEF, low early (microbial) fermentation.

Individual and mean hydrogen excretion data are shown in Figure 1. In 6 patients, H_2_ excretion raised by more than 20 p.m. above the baseline within 1 h after the lactulose ingestion; these individuals were assigned to the High Early microbial Fermentation (HEF) group (Figure 1, red lines). In other 6 patients (Low Early microbial Fermentation–LEF–group; Figure 1, grey lines), >20 ppm H_2_ rise was obtained only after the 60th minute of the test. Hydrogen excretion differed significantly between HEF and LEF groups in the 15th and 30th minute measurements after the lactulose ingestion (p < 0.01, according to the Welch’s t-test).

**FIGURE 2:**
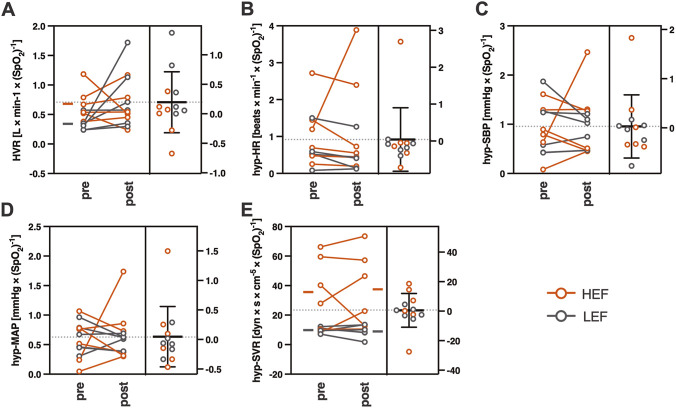
Changes in hypoxic responses before (*pre*) and ∼2 h after (*post*) lactulose test. **(A)** hypoxic ventilatory response, HVR; **(B)** heart rate response to hypoxia, hyp-HR; **(C)** systolic blood pressure response to hypoxia, hyp-SBP; **(D)** mean arterial pressure response to hypoxia, hyp-MAP; **(E)** systemic vascular resistance response to hypoxia, hyp-SVR. For each graph, the left-side part shows individual data (mean values are marked with short lines on margin only if significant HEF vs. LEF difference was obtained), and the right-side part presents the mean of differences (circles represent individual data points; thick line represents mean value; error bars represent ±SD). Data points and lines related to the HEF group are marked with red, and those related to the LEF group are marked with grey. Individual values on graphs **(A–D)** were multiplied by −1 for illustration purposes. HEF, high early (microbial) fermentation; LEF, low early (microbial) fermentation.

### Resting physiological variables and blood glucose level

Resting ventilatory (VI and VT) and haemodynamic (HR, SBP, MAP, SVR) parameters, as well as the blood glucose concentration, were evaluated twice for each participant (before and 2 h after the lactulose ingestion). Mean (±SD) or median values (with lower and upper quartile) of these parameters are summarised in Table 1. No statistically significant changes were obtained, neither for all patients included, nor within the HEF or LEF group (all p > 0.05).

### Hypoxic responses

Hypoxic responses were evaluated before and about 2 h after the lactulose ingestion. Hypoxic ventilatory response (HVR) and hypoxic hemodynamic responses (Hyp-HR, Hyp-SBP, Hyp-MAP, Hyp-SVR) were quantified as the slopes of the linear relationship between SpO_2_ and each given ventilatory or haemodynamic parameter (Table 1).

**FIGURE 3:**
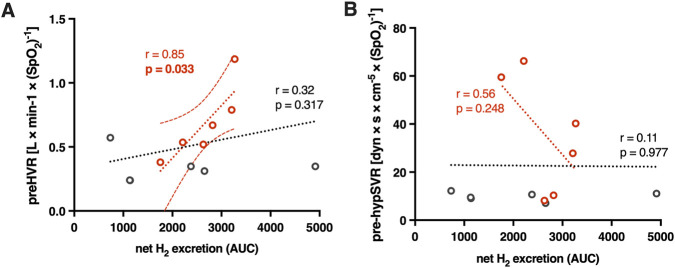
The relation between the pre-lactulose hypoxic ventilatory response (pre-HVR) **(A)** and pre-lactulose systemic vascular resistance to hypoxia (pre-hypSVR) **(B)** and lactulose-induced hydrogen excretion (area under curve of the net H_2_ excretion; net H_2_ AUC). Red circles represent individual data for HEF patients, and grey–for LEF patients. Pearson’s correlation coefficient was used to test the linear association between variables (i.e., red dotted line for HEF, n = 6; black dotted line for all study participants, n = 12); for the HEF subgroup, 95% confidence bands were shown as red dashed lines; r- and p-values are shown above each trend line. Individual values on graph **(A)** were multiplied by −1 for illustration purposes. HEF, high early (microbial) fermentation; LEF, low early (microbial) fermentation.

None of the hypoxic responses changed significantly over the course of experiment (i.e., pre-vs. post-lactulose values; all p > 0.05). Nevertheless, several differences were obtained between HEF and LEF groups as follows: (1) HEF group revealed significantly higher initial (pre-lactulose) HVR than the LEF group (mean ± SD (95%CI), L/min/SpO_2_: 0.680 ± 0.284 (0.38–0.98) vs. 0.343 ± 0.122 (0.22–0.47); p = 0.024, Cohen’s *d* = 1.54); (2) HEF group showed significantly higher pre- and post-lactulose hyp-SVR response values than the LEF group (mean ± SD (95%CI), dyn s/cm^5^/SpO_2_: for pre-lactulose comparison, 35.40 ± 24.41 (9.78–61.01) vs. 9.96 ± 1.80 (8.07–11.85), p = 0.039; Cohen’s *d* = 1.47; for post-lactulose comparison, 37.19 ± 25.75 vs. 9.22 ± 4.33, p = 0.026, Cohen’s *d* = 1.51; Figure 2). According to such differences obtained, we evaluated correlations between ventilatory and SVR hypoxic responses and the magnitude of lactulose-induced hydrogen excretion (area under curve of the net H_2_ excretion; net H_2_ AUC). A positive correlation was found for HEF patients’ pre-lactulose HVR (r = 0.85, p = 0.033; Figure 3A). Noteworthy, the correlation remained significant even under less stringent HEF classification criteria (i.e. 90 min timeframe for the peak H_2_ rise detection), however with a borderline p value (r = 0.74, p = 0.046; Supplementary Figure S1). No such correlations were obtained neither for the pre-lactulose hyp-SVR responses (r = 0.56, p = 0.248; Figure 3B), nor for the post-lactulose HVR or hyp-SVR response values (all p > 0.05).

## Discussion

We have previously demonstrated that the acute increase in gut microbial fermentation augments peripheral chemoreflex sensitivity in young, healthy volunteers (Seredyński et al., 2021). Present study is the first to examine the presence of such interactions among heart failure patients. Individuals exhibiting early (<60 min) rise in gut microbial fermentation after the lactulose ingestion (High Early Fermentation patients, HEF) revealed higher initial ventilatory and vascular responses to hypoxia, compared to the Low Early Fermentation patients (LEF). Moreover, in the HEF group, initial HVR positively correlated with the hydrogen excretion during the lactulose test.

These observations may be interpreted as indicating a link between enhanced bacterial fermentation in the small intestine and heightened peripheral chemosensitivity in HF patients. However, the assessment of microbiota-dependent fermentation using the hydrogen breath test is inherently influenced by inter-individual variability in oro-cecal transit time, which warrants cautious interpretation of these findings. Nevertheless, as discussed in more detail in the *Study Limitations* section, we deliberately applied stringent criteria to define early fermentation in order to virtually eliminate false-positive classification, albeit at the expense of potentially failing to identify some HEF individuals. Notably, applying less conservative criteria did not change the main results of the study (see Supplementary Figure S1).

Among 12 study participants, 6 were classified as HEF, reflecting the prevalence of SIBO reported for larger HF cohorts (Song et al., 2021). Indeed, a growing body of evidence links the gut dysbiosis and disrupted intestinal barrier to worsened inflammation and poor metabolic profiles in HF, potentially aggravating disease progression and mortality (Mollar et al., 2019; Song et al., 2021; Song et al., 2024). In comparison, little is known about the relation between gut microbial disturbances and autonomic imbalance–a hallmark malfunction in HF (Floras and Ponikowski, 2015; Giannoni et al., 2023). Nevertheless, several animal (O’Connor et al., 2019; Wang et al., 2022) and human (Seredyński et al., 2021) studies feature gut microbiota as important modulators of autonomic functions, plausibly via its fermentation-derived metabolites.

In this pilot HF study, we indeed obtained a relation between the gut fermentation intensity and ventilatory response to hypoxia, consistent with the general pattern previously reported in healthy volunteers (Seredyński et al., 2021). It should be emphasized, however, that the two studies differed in terms of experimental protocols and conditions and were conducted independently; therefore, no direct quantitative or statistical comparisons between datasets were performed or intended. Nevertheless, the earlier healthy-volunteer study provides a relevant physiological context for interpreting the present findings. Primarily, our HF cohort did not exhibit a significant overall change in HVR after the lactulose ingestion, in contrast to the lactulose-induced HVR upregulation observed in healthy subjects. Instead, HF patients with increased upper gut fermentation (HEF) showed an intrinsically elevated baseline HVR compared to the LEF group, a difference that disappeared post-lactulose. Moreover, only in the HEF group did hydrogen excretion correlate positively with baseline (but not post-lactulose) HVR, indicating that individuals with greater early gut microbial fermentation tend to have heightened chemoreflex sensitivity even before any experimental intervention.

It is tempting to speculate that early rise in the lactulose-induced hydrogen production (often interpreted as a proxy for bacterial overgrowth of the upper gastrointestinal tract) might be associated with increased small intestinal permeability (Lewis and Taylor, 2020; Kitai et al., 2022), leading to the enhanced outflow of microbial metabolites to the bloodstream (Snelson et al., 2025), which in turn alters the sensitivity of peripheral chemoreceptors. Such hypothesis remains coherent with the lack of direct effect of lactulose ingestion on HVR in HEF group. HEF patients’ ventilatory drive might be already elevated at baseline due to chronic chemoreceptor microbial stimulation, leaving little room for further enhancement with an acute fermentative stimulus. In fact, post-lactulose HVR did not change (or even decreased) for the most of HEF subjects, but raised modestly in LEF patients. Interestingly, rise in HVR following lactulose meal was reported in our previous study in healthy volunteers (Seredyński et al., 2021). However, given the methodological limitations of hydrogen breath testing, such interpretations should be treated with caution. Early hydrogen excretion does not uniquely reflect fermentation capacity and may also be influenced by altered oro-cecal transit. The present findings should be interpreted as identifying an association between hydrogen excretion kinetics and chemoreflex responsiveness, rather than establishing a direct mechanistic link between gut fermentation and PCh function.

Among hemodynamic variables, significant differences were obtained solely for the systemic vascular resistance hypoxic response (hyp-SVR). HEF group revealed higher pre- (baseline) and post-lactulose hyp-SVR values compared to LEF patients. Opposite to HVR, though, we failed to obtain correlation between hyp-SVR and hydrogen excretion. This suggests the small intestinal fermentation affects ventilatory responses in a more direct way that the hemodynamic ones (Seredyński et al., 2021). It aligns with a growing body of data that the two arms of the peripheral chemoreflex can be differentially modulated under the same stimulus (Keir et al., 2019; Zera et al., 2019). Another level of complexity might arise from the bi-directional nature of the gut microbiota-sympathetic division communication. HF itself is known to alter the gut environment: chronic HF leads to intestinal edema and reduced visceral perfusion, which in turn might readjust the vascular sympathetic tone (Fudim et al., 2021), potentially attenuating gastrointestinal motility (Zheng et al., 2024) and promoting gut dysbioses. In line with that, there is rising clinical interest in targeting gut microbial overgrowth for HF management, with a rising number of randomized trials ongoing.

Although direct experimental evidence showing that gut-microbiota modulation can reduce sympathetic activity in HF through alterations in the PCh function is currently lacking, several mechanistic studies provide a physiologically plausible foundation for this pathway. Animal models consistently demonstrate that targeted manipulation of the gut microbiome can attenuate sympathetic overactivity in certain sympathetically-mediated disorders; mostly in hypertension (Klippel et al., 2016; Toral et al., 2019; Kong et al., 2021; Liu et al., 2022; Yin et al., 2024), with more limited but emerging evidence in HF models (Liu et al., 2024). (Klippel et al., 2016; Toral et al., 2019; Kong et al., 2021; Liu et al., 2024; Yin et al., 2024). Reductions in sympathetic activity in these models are typically accompanied by significant improvements in blood pressure, further highlighting the functional relevance of gut–autonomic interactions. These findings are supported by human data showing that interventions that substantially modify the gut environment, i.e., Roux-en-Y gastric bypass (Zhang et al., 2014) or prebiotic supplementation (Jama et al., 2023) can exert measurable antihypertensive effects. On the other hand, well-established role of the heightened peripheral chemosensitivity in generating sympathetic overactivation in HF (Marcus et al., 2014; Marcus et al., 2018; Niewinski et al., 2017; Toledo et al., 2017), and the presence of receptors for microbiota-derived metabolites on the carotid bodies (Peng et al., 2020) provide a plausible cellular substrate through which gut-microbiota alterations could modulate carotid-body signaling and, consequently, autonomic outflow.

Our work has several limitations. First, this was a preliminary observational experiment involving 12 H F patients, without randomization or a control group. As such, the study was not designed to determine causality, and all findings should be interpreted as exploratory. Second, we relied on hydrogen breath tests during the quantification of gut microbial fermentation, as well as classification of participants as HEF or LEF. According to the literature, about 30%–62% of the human population are ‘methane producers’, and in some people methane is even a dominant gaseous product of gut microbial fermentation (De Lacy Costello et al., 2013). Therefore, hydrogen breath tests we performed could lead to the underestimation of the gut fermentation intensity. Nevertheless, hydrogen production has been reported as a better predictor of long-term adverse event risk in HF patients than methane, which supports our focus on hydrogen in this pilot study (Mollar et al., 2019). From a practical standpoint, integrating the lactulose breath test with cardiovascular and autonomic assessments within a single morning visit required a bedside technique with immediate readout; a handheld hydrogen analyzer allowed on-site measurements without gas sample collection or storage, thereby facilitating tight temporal alignment of procedures and minimizing between-session variability. Future studies should employ multi-gas (H_2_/CH_4_) breath testing to more comprehensively characterize fermentation profiles in HF. Moreover, we did not quantify short-chain fatty acids (SCFAs) in blood or feces, so our assessment of fermentation was limited to breath hydrogen; future, larger studies should integrate targeted SCFA/metabolomics profiling to better characterize microbiota-derived metabolites potentially linking gut fermentation with cardiovascular and autonomic regulation.

Thirdly, our HEF/LEF classification relied on an early rise in exhaled hydrogen, an approach that is sensitive to inter-individual variability in oro-cecal transit time (Rangan et al., 2022), which might be of even greater importance in HF with frequent gastrointestinal ailments (Mahenthiran et al., 2024).

Whereas >20 ppm hydrogen cutoff is a recommended value for the lactulose HBT-based detection of the augmented upper gut fermentation in SIBO (Rezaie et al., 2017; Dahlgren et al., 2025), early H_2_ rise timeframes proposed in the literature differ from 60 to 100 min; however, as demonstrated with concomitant scintigraphy, time window >80 min after the lactulose ingestion may substantially increase the risk of false-positive results (Sunny et al., 2016; Dahlgren et al., 2025). As our pilot study did not aim to diagnose SIBO, we deliberately applied very strict lactulose HBT criteria (≥20 ppm rise within 60 min after 10 g lactulose) to focus on an early fermentation phenotype and to minimize the contribution of later, likely colonic fermentation to HEF classification, accepting that this strategy reduces sensitivity and may have led to misclassification of some patients as LEF.

Nevertheless, HEF/LEF classification in this study should be regarded as a pragmatic, hypothesis-generating stratification based on hydrogen kinetics, rather than a definitive marker of intrinsic fermentation capacity.

Finally, as this was a single-centre pilot study, small sample size inevitably limited the statistical power of the between-subgroup comparisons. This limitation also influenced our statistical strategy: although several physiological variables were analysed, we did not apply formal corrections for multiple comparisons. Such procedures (e.g., Bonferroni or Holm) are highly conservative in small mechanistic studies and substantially increase the risk of type II errors, potentially obscuring physiologically meaningful effects. Instead, in line with contemporary statistical recommendations for hypothesis-driven exploratory studies (Perneger, 1998; Armstrong, 2014), we prioritised the interpretation of effect sizes and confidence intervals around the means. Notably, for the three key findings of the study, effect sizes quantified as Cohen’s *d* exceeded 1.4, which is conventionally interpreted as a large effect, supporting the physiological relevance of these observations despite limited sample size (Cohen, 1992). On the other hand, the study population was somewhat homogenous (in terms of sex, NYHA class, pharmacological treatment, etc.), and several precautions were taken to minimize the intrasubject variability (all data–H_2_ excretion, resting parameters, hypoxic responses–were collected during one morning session after the overnight fasting). Nevertheless, further controlled trials in a larger cohort are warranted.

Present findings demonstrate that increased upper gut fermentation in HF may contribute to the augmented hypoxic responsiveness prior to any acute challenge. This extends the findings from healthy subjects by indicating that intrinsic gut microbial activity might be an additional factor shaping peripheral chemosensitivity in HF. Unravelling this putative gut–peripheral chemoreflex connection may broaden our understanding of HF pathophysiology, but given the exploratory, non-randomized design and small sample size, these observations should be viewed as hypothesis-generating and require validation in larger cohorts before any clinical implications can be drawn.

## Data Availability

The raw data supporting the conclusions of this article will be made available by the authors, without undue reservation.
