# Supplementary material for: Hypoxic responsiveness and gut fermentation capacity in heart failure patients: preliminary results
Source: Front Physiol. 2026 Jan 8;16:1728753. doi: 10.3389/fphys.2025.1728753 (PMC12823947; doi:10.3389/fphys.2025.1728753)
Supplement: Supplementary file 1 [file Table1.docx]

**SUPPLEMENTARY MATERIAL**

**Hypoxic responsiveness and gut fermentation capacity in heart failure patients:
preliminary results**

Aleksandra Mikołajczak^,^ Rafał Seredyński, Marzena Gonerska, Mateusz Sokolski,

Bartłomiej Paleczny, Beata Ponikowska


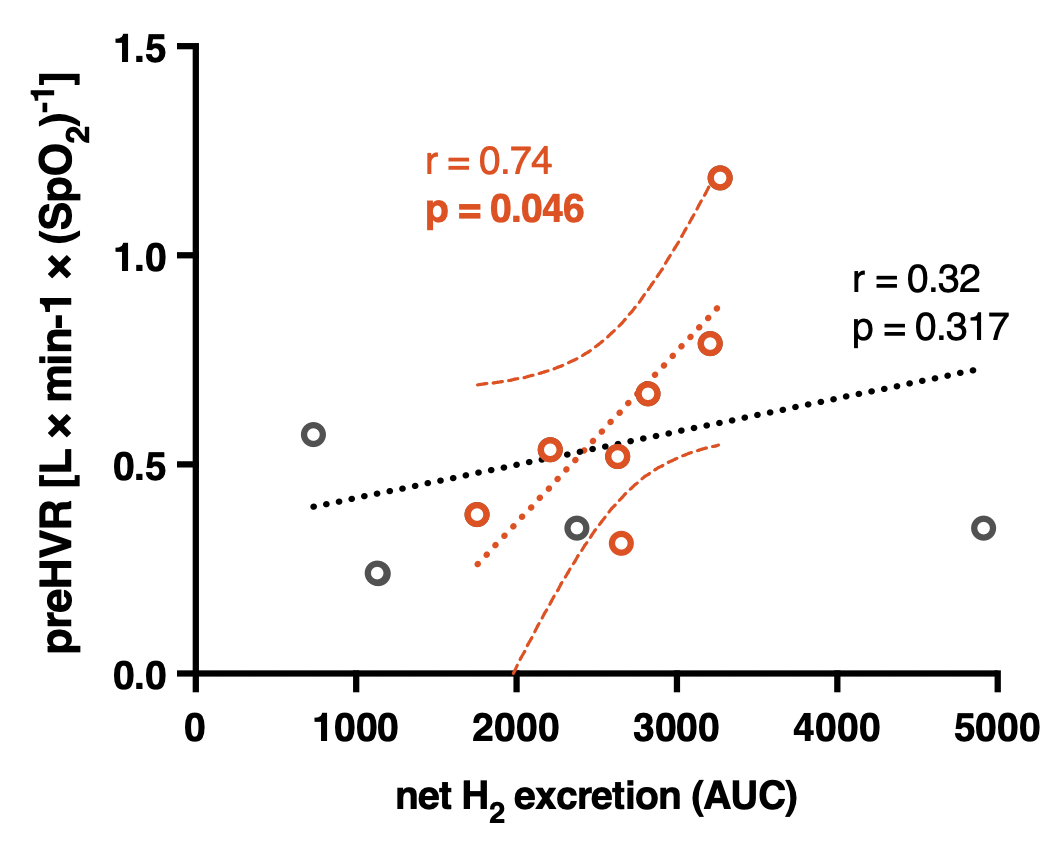


**Supplementary Fig. 1.** The relation between the pre-lactulose hypoxic ventilatory response (pre-HVR) and lactulose-induced hydrogen excretion (area under curve of the net H_2_ excretion; net H_2_ AUC), obtained for an alternative HEF classification criterion (H_2_ rise detection timeframe extended from 60 to 90 min compared to Fig. 3). Red circles represent individual data for HEF patients, and grey – for LEF patients. Pearson’s correlation coefficient was used to test the linear association between variables (i.e. red dotted line for HEF, n = 7; black dotted line for all study participants, n = 12); for the HEF subgroup, 95% confidence bands were shown as red dashed lines; r- and p-values are shown above each trend line. Individual values on graph (a) were multiplied by −1 for illustration purposes. HEF, high early (microbial) fermentation; LEF, low early (microbial) fermentation.
